# Psychometric validation of the Chinese versions of the quality of communication questionnaires for cancer patients and their family caregivers

**DOI:** 10.1186/s12912-024-02071-z

**Published:** 2024-06-20

**Authors:** Zhihan Chen, Yanjia Li, Zhishan Xie, Siyuan Tang, Jinnan Xiao

**Affiliations:** Xiangya School of Nursing, Central South University, NO. 172 Rd, Changsha, 410013 China

**Keywords:** Communication, Cancer patients, Family caregivers, Cultural adaptation, Psychometric property, Validation

## Abstract

**Background:**

Given the lack of valid and reliable instruments for evaluating the quality of communication between physicians and cancer patients and their family caregivers in China, this study translated and culturally adapted the Quality of Communication questionnaires for cancer patients (QOC-P) and their family caregivers (QOC-F) for use in the Chinese context and evaluated their psychometric properties.

**Methods:**

The QOC-P and QOC-F were translated following an adapted version of Brislin’s translation model and culturally adapted according to a Delphi expert panel. We pretested and refined the Chinese versions of the QOC-P and QOC-F among 16 dyads of patients and their family caregivers. Subsequently, we administered the questionnaires to 228 dyads of patients and their family caregivers who were recruited from six tertiary hospitals. The content validity, construct validity, convergent validity, and reliability of the QOC-P and QOC-F were examined.

**Results:**

Through exploratory factor analysis, The QOC-P and QOC-F were divided into two dimensions: general communication and end-of-life communication. The Cronbach’s coefficients ranged from 0.905 to 0.907 for the two subscales of the QOC-P and from 0.908 to 0.953 for the two subscales of the QOC-F. The two-week test-retest reliability was acceptable for both the QOC-P and QOC-F, with intraclass correlation coefficients of 0.993 and 0.991, respectively. The scale content validity index (QOC-P: 0.857, QOC-F: 1.0) and split-half reliability (QOC-P: 0.833, QOC-F: 0.935) were satisfactory. There was a negative correlation with anxiety and depression for both the QOC-P (*r* = -0.233 & -0.241, *p* < 0.001) and QOC-F (*r* = -0.464 & -0.420, *p*<0.001). The QOC-P showed a negative correlation with decision regret (*r* = -0.445, *p*<0.001) and a positive correlation with shared decision-making (*r* = 0.525, *p*<0.001), as hypothesized.

**Conclusion:**

The QOC-P and QOC-F show acceptable psychometric properties for evaluating the quality of communication between physicians and cancer patients and their family caregivers in both clinical and research contexts. Future studies should use more diverse and inclusive samples to test the structure of the Chinese version of the QOC-P and QOC-F with confirmatory factor analysis.

**Supplementary Information:**

The online version contains supplementary material available at 10.1186/s12912-024-02071-z.

## Background

According to the International Agency for Research on Cancer, an estimated 19.3 million new cancer cases and almost 10 million cancer deaths occurred worldwide in 2020 [1]. Despite continuous developments in medical technology, the prevalence and mortality of cancer are continuing to rise [2]. Furthermore, the global cancer burden is expected to reach 28.4 million cases in 2040 – a 47% rise from 2020 [1]. The high incidence of cancer and the complex, varied, high-risk treatment options trigger difficult medical decisions for cancer patients and their family caregivers. Despite the continuous development of medicine and the increasing variety of therapeutic modalities, the effects of different treatment options are still uncertain, the best treatment option is difficult to determine [3, 4]; On the other hand, the limited medical knowledge of cancer patients, the small amount of time for doctors to communicate with cancer patients and their family caregivers about medical decision-making, the fact that cancer patients are facing with a variety of physical and psychological symptoms and the high cost of cancer treatment, these consequently exacerbate the communication dilemma between the three parties [5–7]. The importance of physician-patient communication through associations between physician-patient communication and positive patient health outcomes have fully demonstrated and confirmed [8], including easier decision-making [9], better medical adherence and patient emotional health [10], and stronger physician-patient relationships [11], which in turn increase patient satisfaction with care.

Physician-family communication is also key for improving healthcare, especially in countries and areas that have long been influenced by family culture, such as China. In these places, the family has a predominant role in communicating with physicians on the patient’s behalf to, for instance, receive diagnostic information or make treatment decisions [12]. Research has demonstrated that a higher quality of physician-family communication can help family caregivers develop a better understanding of a patient’s medical condition and prognosis or the effectiveness of treatments [13]. Moreover, it can build trust, alleviate anxiety, and support structured communication with healthcare providers [14].

In China, the status of communication between physicians and their patients as well as their patients’ family members is not satisfactory [15]. A recent review by [16] has confirmed a steady increase in medical violence in China from 2013 to 2016, which seriously affects the physician-patient relationship and is indicative of poor communication between physicians and patients. In China, the shortage of physicians compared to the high number of patients has exacerbated problems in communication quality between physicians and their patients as well as their patients’ family caregivers. Furthermore, there is an uncomprehensive communication about end-of-life care between patients and physicians. Although hospice care was introduced in Mainland China in the late 1980s, public awareness remains low, and there is a gap in communication about end-of-life care in the Chinese context [17]. Therefore, to promote the quality of communication between physicians and patients as well as their family caregivers is essential.

Systematic assessment is the first step toward improving communication. The Quality of Communication questionnaire was originally developed in 2004 to assess English-speaking patients’ perceptions of the quality of their communication with their physicians [18]. The items for this Quality of Communication questionnaire for patients (QOC-P) were generated from a series of qualitative studies among patients with chronic obstructive pulmonary disease (COPD), cancer, and acquired immunodeficiency syndrome (AIDS) [18]. Later, a Quality of Communication questionnaire for family caregivers (QOC-F) was developed to measure family members’ perceptions of the quality of their communication with physicians or nurse practitioners [19]. The QOC-P has been used among patients with AIDS, cancer, and COPD [20, 21]. The QOC-P has been translated and culturally adapted for use in other countries, such as Brazil [22] and Italy [23], and the QOC-F has been translated and culturally adapted for the Korean context [13]. These studies suggest that the QOC-P and QOC-F are reliable instruments for evaluating communication between physicians and their patients as well as their patients’ family caregivers.

In China, there is a lack of standardized instruments for assessing concepts associated with the quality of communication between physicians and patients and their family caregivers. Therefore, this study aimed to translate and culturally adapt the QOC-P and QOC-F for use in the Chinese context and evaluate their psychometric properties for use among cancer patients and their family caregivers according to the content validity, construct validity, convergent validity, and reliability.

## Methods

In the present study, the QOC-P and QOC-F were translated into Chinese and adapted to measure the quality of communication between physicians and cancer patients and their family members in China. The original author approved the translation and adaptation of QOC-P and QOC-F for use in China via email. The study was approved by the ethics committee of our university and performed in accordance with the established ethical standards.

### Translation and cultural adaptation

The QOC-P and QOC-F were translated into Chinese and adapted for use in China following an adapted version of Brislin’s translation model [24]. Initially, two Chinese translators who have a good command of English independently translated the original versions of the QOC-PF into Chinese. One translator was a master’s student majoring in English, and the other was an oncology researcher who was proficient in English and had studied abroad in Australia for three years. The translated versions were designated T1 and T2 and a synthesis of T12 was produced through internal discussion. Subsequently, two translators independently back-translated T12 into English who have proficiency in English and unfamiliar with the original QOC-PF. One translator was majoring in English, and the other was an oncology professional who was proficient in English and Chinese. The back-translated versions were named BT1 and BT2. An expert with extensive experience in palliative care who was fluent in English was invited to compare BT1 and BT2 with the original versions of the QOC-P and QOC-F. Based on the expert’s feedback, further revisions were made to T12.

To achieve semantic, idiomatic, conceptual, and cultural equivalence of the T12 at that point, an expert panel was formed with four academic researchers in palliative care or oncology nursing, three oncology nurses, and one nursing manager. The committee members independently assessed the semantic, empirical, and conceptual equivalence of T12 to the original texts. Based on their assessments, our team discussed and adjusted T12 to arrive at the “prefinal” versions of the QOC-P and QOC-F for use in China. These versions were used in the pretesting phase of the study, which included 16 dyads of cancer patients and their family caregivers. In the pretesting phase, participants were asked for comments on items that were rated as unclear or culturally inappropriate to improve the clarity and cultural appropriateness of them. After analyzing all the participants’ responses and suggestions, our team made adjustments and prepared the final versions of the QOC-P and QOC-F for use in China.

### Participants

This study employed a convenience sampling method to select patients with cancer who were being treated or hospitalized at six tertiary hospitals and their family caregivers in Hunan Province from June to December 2023. As the inclusion criteria, the cancer patients had to be 18 years of age or older, have a known diagnosis of cancer, and have a family caregiver who also agreed to take part in the study. The exclusion criteria for cancer patients were being in a coma, having a neurological or psychiatric disorder, presenting with hearing loss or any other condition affecting communication, and using medications that can alter the level of consciousness.

Family caregivers of adult cancer patients were invited to participate if they were 18 years of age or older and self-identified as a primary caregiver (i.e. the family caregiver who was most likely to provide pre-admission or post-discharge caregiving or who was most involved in the patient’s treatment decisions). The exclusion criteria for cancer patients’ family caregivers were having mental health, and severe medications dependence that can alter the level of consciousness.

To conduct EFA analysis, at least 200 samples are needed [3]. To calculate the sample size for a cross-sectional study, we used the following formula identified by [3] cross-sectional research in medical statistics: 19 * (5–10) * 1.2 = 114–228. Comprehensively considering the aim of our study, we finally confirmed that the sample size of our study was 228. Therefore, 228 the Chinese version of the QOC-P questionnaires were distributed to cancer patients and 228 the Chinese version of the QOC-F questionnaires were distributed to their family caregivers during the survey period. A total of 219 valid questionnaires were recovered respectively from cancer patients and their family caregivers, which represents a valid recovery rate of 96.1%.

### Instruments

General demographic information and clinical data were collected for gender, age, educational level, marital status, occupational status, weight loss during treatment, diagnosis, and stage of disease. The clinical data were reported by the participants or retrieved from their medical records.

### The QOC-P and QOC-F

The QOC-P and QOC-F were developed [18] to assess the perceptions of patients and their family caregivers, respectively, regarding the quality of their communication with physicians. The QOC-P contains 19 items, and the QOC-F contains 15 items. Each questionnaire covers two dimensions: general communication skills and communication skills about end-of-life care. For all items, communication quality is rated on a scale from 0 (poor) to 10 (absolutely perfect), participants were offered two additional response options: “Physician or nurse did not do this” and “I do not know.” Higher scores indicate a better perceived quality of communication. The Brazilian version of the QOC-P has shown acceptable internal consistency (Cronbach’s α > 0.75) among patients in intensive care and palliative care [21], while the Korean version of the QOC-F has shown acceptable internal consistency (Cronbach’s α ≥ 0.85) among family caregivers of ICU patients [13]. The final Chinese version of the QOC-P and QOC-F were provided in the “supplementary file-the final scales”.

### Chinese version of the patient health questionnaire for depression and anxiety

The Patient Health Questionnaire for Depression and Anxiety (PHQ-4) was developed [25] as a valid ultra-brief tool for detecting anxiety and depressive disorders. In our study, we used the Chinese version of the PHQ-4, which was translated by [26] and consists of four items measuring the emotional state of patients over the past two weeks. The first two items relate to depression, while the latter two target anxiety. Each item is rated on a four-point scale (0 = completely unknown, 1 = a few days, 2 = more than half of the days, 3 = almost every day). With a Cronbach’s alpha of 0.833, the Chinese version of the PHQ-4 has shown good reliability among Chinese patients [26].

### Chinese version of the decision regret scale

The Decision Regret Scale (DRS) is a valid and reliable instrument for individual self-assessment of regret level about a health-related decision. In our study, we used the Chinese version of the DRS (DRS-C), which was translated by [27]. The DRS-C contains five items and uses a five-point rating system ranging from 1 (strongly agree) to 5 (strongly disagree). The scores are reversed for Items 2 and 4. Mean scores are obtained and then converted by subtracting 1 and multiplying by 25. Internal consistency reliability estimates (a) range from 0.81 to 0.92 among oncology patients [28].

### Chinese version of the nine-item shared decision making questionnaire

The nine-item Shared Decision Making Questionnaire (SDM-Q-9) [29] is a self-assessment tool for measuring the degree to which patients and doctors participate in collaborative decision-making. The SDM-Q-9 has been translated into Chinese [30]. The questionnaire uses a five-point Likert scoring method, and the total score ranges from 0 to 45. For comparison purposes, the original authors have suggested converting the original score by * 20/9 to 0 to 100 points. With a Cronbach’s alpha of 0.945, the Chinese version of the SDM-Q-9 has shown good reliability among Chinese patients [30].

### Data collection

After securing ethical approval, eligible patients were approached for this study, meanwhile, each participant signed written consent form. Patients and their family caregivers independently filled out the questionnaires under the guidance of a research assistant within a period of 15 to 20 min. The QOC-P and QOC-F were administered again two weeks later among a convenient subsample to evaluate the test-retest reliability of the instruments. A total of 20 pairs of cancer patients and their family caregivers could be reached in wards to complete the second test.

### Data analysis

Our data were analyzed using SPSS 26.0 version and AMOS 24.0 version. First, we used Shapiro-wilk test to conduct normality test. If *p* > 0.05, which indicated that our data were normally distributed, we would use the mean, standard deviation (SD), and frequency (%) to describe the study sample and the scores of questionnaires. If *p* < 0.05, which indicated that our data were skew distribution, we would use the median, quartile, 95% confidence interval to describe the study sample and the scores of questionnaires. Standard error of measurement (SEM) for the Chinese version of the QOC-P and QOC-F were calculated. We also evaluated Minimal clinical important difference (MCID, MCID = 1.96*√2*SEM) for the QOC-P and QOC-F to reflect the minimal change in score considered relevant by cancer patients-their family caregivers and physicians [31]. We conducted confirmatory factor analysis (CFA) and exploratory factor analysis (EFA) to explore the structure validity of the Chinese version of the QOC-P and QOC-F, conducted the Pearson correlation to explore convergent validity, and calculated Cronbach’s alpha coefficient, item-to-total correlation, composite reliability (CR), and intraclass correlation coefficient (ICC) to explore reliability.

### Content validity

We calculated the content validity index (CVI) to reflect content validity, which comprising item CVI (I-CVI) and scale CVI (S-CVI). Our expert panel was invited to evaluate the content validity of the QOC-P and QOC-F on a four-point scale (1 = not relevant, 2 = somewhat relevant, 3 = quite relevant, 4 = highly relevant). The I-CVI was calculated as the number of experts who gave a rating of 3 or 4 divided by the total number of raters, while the S-CVI was computed by averaging all I-CVIs. Minimum values of 0.8 for the I-CVI and 0.9 for the S-CVI were considered acceptable.

### Structural validity

The two dimensions of the original version of the QOC-P and QOC-F have been well confirmed in Brazil, Italy, and Korean. In our study, we firstly conducted CFA using 219 pairs of cancer patients and their family caregivers’ data based on the two dimensions of the original version of the QOC: general communication skills and communication about end-of-life care. Degree of freedom ratio (2 < CMIN/DF < 3), root mean square error of approximation (RMSEA < 0.08), and comparative fit index (CFI > 0.9) were used to determine model fitness. If CMIN/DF > 3 and CFI < 0.9 in the Chinese version of the QOC-P and QOC-F, we need re-explore the dimension of the scale using the same data by first calculating the Kaiser-Meyer-Olkin sampling fitness measure of the scale and applying the Bartlett’s spherical test to determine whether the scale was suitable for EFA. Kaiser-Meyer-Olkin values fluctuate from 0 to 1, and a higher value indicates a better effect of the factor analysis. In our study, the cumulative variance contribution of the final extracted common factors needed to be greater than 50%, and each entry had a high factor loading (> 0.50) on its common factor. If the entry’s factor loading < 0.50, the entry was deleted in our study.

### Convergent validity

For the convergent validity, we used the Pearson correlation to determine the correlation of the QOC-F with the score for depression and anxiety, and the correlation of the QOC-P with the score for decision regret, shared decision-making, depression and anxiety. We hypothesized a negative correlation between the communication quality (among physicians and cancer patients-their family caregivers) and depression and anxiety. We also hypothesized that communication quality between physicians and patients was negatively associated with decision regret and positively associated with shared decision-making.

Convergent validity was also evaluated based on Average Variance Extracted (AVE) value. AVE can be calculated by the formula AVE= (∑λ^2^)/n, (n is the number of topics in a factor; λ is the factor loading value). AVE reflects how much of the variance explained by each latent variable comes from all the topics in the latent variable, and when the AVE value is greater than 0.50 it means that the latent variable has a better convergent validity.

### Reliability

We evaluated the reliability of the QOC-P and QOC-F by examining the instruments’ internal consistency and test-retest reliability. Internal consistency was determined by the Cronbach’s α coefficient and item-to-total correlation. At the same time, we evaluated composite reliability (CR) to reflect the internal consistency. Minimum values of 0.7 for the Cronbach’s alpha and CR, and 0.4 for the item-to-total correlation were adopted. An ICC value of less than 0.4 represents poor test-retest reliability, a value between 0.4 and 0.75 signifies fair to good reliability, and a value above 0.75 denotes excellent reliability.

## Results

### Participant characteristics

Through normality test, our data presented an approximate normal distribution (see Table S1 and S2 in the supplementary file), therefore we used mean, SD, and frequence (%) to describe our study sample and the scores of questionnaires. The mean (SD) age of patients was 58.13 ± 10.908 years. Approximately 60% were women (*n* = 124, 56.6%), and approximately 60% were married (*n* = 207, 94.5%). Breast and gynecological tumors were most common forms of our sample (*n* = 101, 46.1%). The vast majority of patients had been diagnosed within six months (*n* = 179, 81.7%). Most were in the second or third stage of cancer (*n* = 149, 68.1%) and being treated with radiotherapy and chemotherapy (*n* = 139, 62.6%). The mean (SD) age of the family caregivers was 46.79 ± 11.990. Approximately 55% were women (*n* = 122, 55.7%), and half had completed at least a high school education (*n* = 110, 50.2%). The majority were an adult child (*n* = 93, 42.5%) or the spouse (*n* = 100, 45.7%) of the patient. The general information of participants is performed in Table 1.

**Table 1:** General information of research subjects (*n* = 219)

Patients	*N*	%	Caregivers	*N*	%
**Age**			**Age**		
≤ 44	22	10.0	≤ 44	99	45.2
45–59	94	42.9	45–59	86	39.3
≥ 60	103	47.0	≥ 60	34	15.5
**Gender**			**Gender**		
Male	95	43.4	Male	97	44.3
Female	124	56.6	Female	122	55.7
**Nationality**			**Nationality**		
Han	217	99.1	Han	215	98.2
other	2	0.9	other	4	1.8
**Occupation**			**Occupation**		
Students	2	0.9	Students	3	1.4
Farmers	71	32.4	Farmers	30	13.7
Industry and mining	63	28.8	Industry and mining	51	23.3
Education	8	3.7	Education	10	4.6
Health	1	0.5	Health	1	0.5
Businessman	15	6.8	Businessman	56	25.6
Other	59	26.9	Other	68	31.1
**Marital status**			**Marital status**		
Unmarried	7	3.2	Unmarried	18	8.2
Married	207	94.5	Married	199	90.9
Widowed	5	2.3	Divorced	1	0.5
			other	1	0.5
**Education**			**Education**		
Graduate and upper level	1	0.5	Graduate and upper level	2	0.9
Undergraduate	11	5.0	Undergraduate	32	14.6
Junior college	7	3.2	Junior college	21	9.6
Senior school	84	38.4	Senior school	110	50.2
Middle school	82	37.4	Middle school	41	18.7
Primary school	34	15.5	Primary school	13	5.9
Religious belief			Religious belief		
Yes	15	6.8	Yes	13	5.9
No	204	93.2	No	206	94.1
**Monthly income(yuan)**			**Relationship**		
< 1000	5	2.3	Spouse	100	45.7
1000–2999	33	15.1	Children	93	42.5
3000–4999	92	42.0	Sibling	14	6.4
5000–9999	81	37.0	Other	12	5.5
≥ 10,000	8	3.7			
**Medical insurance**					
Self-funded	5	2.3			
New rural cooperative medical	83	37.9			
Resident medical insurance	54	24.7			
Employee medical insurance	74	33.8			
Other	3	1.4			
**Children status**					
None	8	3.7			
One daughter	36	16.4			
One son	60	27.4			
One more child	115	52.5			
**Disease type**					
Lung cancer	40	18.3			
Breast and gynecological tumors	101	46.1			
Gastrointestinal cancer	15	6.8			
Lymph cancer	17	7.8			
Head and neck cancer	31	14.2			
Urinary system cancer	4	1.8			
Other	11	5.0			
**Diagnosis time**					
< 6 months	179	81.7			
6–12 months	17	7.8			
1–5 years	11	5.0			
> 5 years	12	5.0			
Stages of disease					
I	18	8.2			
II	84	38.4			
III	65	29.7			
IV	41	18.7			
Not clear	11	5.0			
**Treatment methods**					
Chemoradiotherapy	137	62.6			
Operation	19	8.7			
Chemoradiotherapy & operation	45	20.5			
Drug	7	3.2			
Other	11	5.1			

### The score of each item and total score of the QOC-P and QOC-F

In the Chinese version of the QOC-P, the item “Involving your loved ones in decisions about your illness and treatment” had highest average score, at 9.12 (SD = 1.258). Conversely, the item “Involving you in treatment decisions that you want if you get too sick to speak for yourself” had lowest average score, which was 7.26 (SD = 1.998). The average total score of the QOC-P was 82.40 (SD = 10.33), and SEM for the QOC-P was 0.698, and MCID for the QOC-P was 1.935.

In the Chinese version of the QOC-F, the item “Involving you in discussions about the illness and treatment of your loved one” had highest average score, standing 9.30 (SD = 1.194). While the item “Asking about your spiritual or religious beliefs” had lowest average score, at 7.01 (SD = 2.589). The average total score of the QOC-F was 84.11 (SD = 10.93), and SEM for the QOC-F was 0.739, and MCID for the QOC-F was 2.048. The detailed information on individual item score was performed in the Tables 2 and 3.

**Table 2:** The individual item score and total score of the Chinese version of the QOC-P

Item	Mean	SD
General communication skills	67.53	8.453
1. Using words that you can understand.	8.44	1.265
2. Looking into your eyes.	8.38	1.234
3. Involving your loved ones in decisions about your illness and treatment.	9.12	1.258
4. Answering all your questions about your illness and treatment.	8.67	1.105
5. Listening to what you have to say.	8.39	1.305
6. Caring about you as a person.	8.12	1.322
7. Giving you his/her full attention.	8.18	1.311
8. Talking to you about the details of how you might get sicker.	8.94	1.142
Communication about end-of-life care	42.83	15.610
9. Talking with you about your feelings regarding the possibility that you might get sicker.	8.52	1.286
10. Talking to you about how long you might have to live.	7.47	1.998
11. Involving you in treatment decisions that you want if you get too sick to speak for yourself.	7.26	1.998
12. Asking about things that are important to you in your life.	7.47	1.945
13. Respecting things that are important to you in your life.	7.87	1.408
14. Asking about your spiritual or religious beliefs.	7.47	1.578
15. Respecting your spiritual or religious beliefs.	7.63	1.471
Total	82.40	10.33

**Table 3:** The individual item score and total score of the Chinese version of the QOC-F

Item	Mean	SD
General communication skills scale	52.08	7.085
1. Using words that you can understand.	8.55	1.277
2. Providing you with information about your loved one’s illness and treatment.	9.15	1.084
3. Involving you in discussions about the illness and treatment of your loved one.	9.30	1.194
4. Answering all questions about the illness and treatment of your loved one.	8.77	1.115
5. (The doctor) Talking with you about the detail that your loved one’s disease.	9.04	1.037
6. Helping your family in deciding what kind of treatment your loved one wants.	8.35	1.284
Communication about end-of-life care scale	49.18	10.254
7. Listening to what you have to say.	8.19	1.403
8. Caring about you as a person.	7.74	1.543
9. Giving you his/her full attention.	7.70	1.549
10. When your loved one is able to speak for himself/herself, (the doctor) ask what kind of treatment he/she wants.	8.48	1.184
11. (The doctor) talking with you about your feelings that your loved one might get sicker or die.	8.49	1.174
12. Asking you about things that are important to your loved one in his/her life.	7.60	1.814
13. Asking about your spiritual or religious beliefs.	7.01	2.589
Total	84.11	10.93

### Review of cultural adaptation

During the committee’s review of the translated versions of the original QOC-P and QOC-F, some changes were made to the QOC-P. Item “Talking to you about how long you might have to live,” was considered overly blunt in its expression of death for Chinese culture. “Talking about your life expectancy” was recommended instead. In addition, we deleted Item “Talking to you about what dying might be like,” and Item “Talking to your loved ones about what your dying might be like.” Fourteen out of 16 patients indicated that these sentences made them very uncomfortable, and nearly half (7 out of 16) reported crying, feeling depressed, and being unwilling to fill them out. Based on this feedback and expert discussions, we decided that these two items were not suitable for inclusion in the Chinese cultural context.

Similarly, all 16 family caregivers felt strongly uncomfortable and dissatisfied with the two items “Talking with you about when and how your loved one might get sicker or die” and “Talking to you about how long your loved one might get sicker or die” on the QOC-F. After discussion within our research team, these two items were replaced by the item “Talking to you about the details concerning the possibility that your loved one might get sicker (such as life expectancy, treatment, prognosis, etc.)” on the Chinese version of the QOC-F. The final Chinese version of the QOC-P and QOC-F are presented in Table S3 and Table S4 in the supplementary file.

### Content validity of the QOC-P and QOC-F

In this study, seven experts were invited to evaluate the Chinese versions of the QOC-P and QOC-F. The number of experts who provided a score of 3 or 4 was counted. The I-CVI for the QOC-P was calculated to be 0.857, and the S-CVI was 0.977 for the QOC-P. The I-CVI for the QOC-F was calculated to be 1, and the S-CVI was 1 for the QOC-F. This indicates that the QOC-P and QOC-F have satisfactory content validity.

### Structural validity of the QOC-P and QOC-F

The CFA results indicate that the original two-factor structure of the QOC-P and QOC-F was not unsatisfactory (QOC-P: CMIN/DF = 7.841, CFI = 0.738, RMSEA = 0.000; QOC-F: CMIN/DF = 9.209, CFI = 0.796, RMSEA = 0.000). Through EFA, we identified two factors for QOC-P and QOC-F, though with different constituting items compared to the findings of the instrument developers. Specifically, on the QOC-P, the item “Talking to you about the details of how you might get sicker” was classified in the general communication skills dimension, and the item “Talking with you about your feelings regarding the possibility that you might get sicker” was classified in the communication about end-of-life care dimension.

On the QOC-F, the items “(The doctor) talking with you about the details of your loved one’s disease” and “Helping your family in deciding what kind of treatment your loved one wants” were classified in the general communication skills dimension, whereas “Listening to what you have to say” and “Caring about you as a person” were classified in the communication about end-of-life care dimension. More details are presented in Tables 4 and 5.

**Table 4:** Exploratory Factor Analysis of the Chinese version of the QOC-P

The Chinese version of the QOC-*P* domains	Factors loadings	
	Factor 1	Factor 2
General communication skills		
1. Using words that you can understand.	0.861	
2. Looking into your eyes.	0.895	
3. Involving your loved ones in decisions about your illness and treatment.	0.776	
4. Answering all your questions about your illness and treatment.	0.862	
5. Listening to what you have to say.	0.876	
6. Caring about you as a person.	0.690	
7. Giving you his/her full attention.	0.682	
8. Talking to you about the details of how you might get sicker.	0.646	
**Communication skills about end-of-life care**		
9. Talking with you about your feelings regarding the possibility that you might get sicker.		0.818
10. Talking to you about how long you might have to live.		0.715
11. Involving you in treatment decisions that you want if you get too sick to speak for yourself.		0.557
12. Asking about things that are important to you in your life.		0.880
13. Respecting things that are important to you in your life.		0.729
14. Asking about your spiritual or religious beliefs.		0.889
15. Respecting your spiritual or religious beliefs.		0.766

**Table 5:** Exploratory Factor Analysis of the Chinese version of the QOC-F

The Chinese version of the QOC-F domains	Factors loadings	
	Factor 1	Factor 2
General communication skills		
1. Using words that you can understand.	0.845	
2. Providing you with information about your loved one’s illness and treatment.	0.902	
3. Involving you in discussions about the illness and treatment of your loved one.	0.873	
4. Answering all questions about the illness and treatment of your loved one.	0.715	
5. (The doctor) Talking with you about the detail that your loved one’s disease.	0.614	
6. Helping your family in deciding what kind of treatment your loved one wants.	0.707	
**Communication skills about end-of-life care**		
7. Listening to what you have to say.		0.766
8. Caring about you as a person.		0.827
9. Giving you his/her full attention.		0.824
10. (The doctor) Talking with you about your feelings that your loved one might get sicker or die.		0.853
11. (The doctor) Talking with you about the detail that your loved one’s disease		0.816
12. Asking you about things that are important to your loved one in his/her life.		0.876
13. Asking about your spiritual or religious beliefs.		0.823

### Convergent validity of the QOC-P and QOC-F

There was a negative correlation with anxiety and depression for both the QOC-P (*r* = -0.233 & -0.241, *p* < 0.001). Additionally, the QOC-P was negatively correlated with the degree of decision regret (*r* = -0.445, *p*<0.001) and positively correlated with the degree of shared decision-making (*r* = 0.525, *p*<0.001), as hypothesized. In the QOC-P, the AVE for the general communication skills dimension was 0.3766, the AVE for the communication about end-of-life care dimension was 0.3611.

The QOC-F was negatively correlated with anxiety and depression (*r* = -0.464 & -0.420, *p*<0.001), as hypothesized. In the QOC-F, the AVE for the general communication skills dimension was 0.5107, the AVE for the communication about end-of-life care dimension was 0.3369.

### Reliability of the QOC-P and QOC-F

The Cronbach’s alpha coefficient for the Chinese version of the QOC-P scale was 0.937, and the Cronbach’s alpha coefficients of the dimensions ranged from 0.905 to 0.907. The split-half reliability of the Chinese version of the QOC-P was 0.833. The results show that the test-retest reliability values of the QOC-P was 0.993. In the QOC-P, the CR for the general communication skills dimension was 0.8038, the CR for the communication about end-of-life care dimension was 0.7942.

For the Chinese version of the QOC-F scale, the Cronbach’s alpha coefficient was 0.967, and the split-half reliability was 0.935. The questionnaire was retested after two weeks among 20 pairs of cancer patients and their family caregivers. The results show that the test-retest reliability values of the QOC-F was 0.991. In the QOC-F, the CR for the general communication skills dimension was 0.7530, the CR for the communication about end-of-life care dimension was 0.7428. More detailed information is provided in Table 6.

**Table 6:** Reliability of the Chinese version of the QOC-P and QOC-F scales and subscales

QOC	Number of items	Cronbach’s α	Split-half reliability	CR	ICC
QOC-P	15	0.937	0.833		0.993
General communication skills	8	0.907	0.853	0.8038	
Communication skills about end-of-life care	7	0.905	0.895	0.7942	
QOC-F	13	0.967	0.935		0.991
General communication skills	6	0.908	0.862	0.7530	
Communication skills about end-of-life care	7	0.953	0.884	0.7842	

For the QOC-P, inter-item correlations ranged from 0.104 to 0.912 and the item-to-total correlations ranged from 0.508 to 0.850. For the QOC-F, inter-item correlations ranged from 0.475 to 0.946, and item-to-total correlations ranged from 0.726 to 0.915.

## Discussion

This study represents the inaugural effort to translate and adapt the QOC-P and QOC-F for application within the Chinese cultural milieu. Furthermore, we rigorously examined and deliberated upon the psychometric characteristics of these assessment tools. Our findings unequivocally affirm that the Chinese versions of the QOC-P and QOC-F exhibit commendable content validity, convergent validity, and reliability. Consequently, we posit that these instruments are well-suited for assessing the quality of communication between physicians and cancer patients, as well as their family caregivers, in both clinical and research settings in China.

In the Chinese versions of the QOC-P and QOC-F scales, we omitted items related to death, such as “Discussing what dying might be like with you” and “Discussing with your loved ones what dying might be like for you.” Although we acknowledge that these removed items are integral to the original QOC-P and QOC-F scales, they are presently unsuitable for implementation within the cultural context of China. Findings from the preliminary test indicate that Chinese cancer patients and their family caregivers exhibited heightened sensitivity towards the progression of the illness and the imminent prospect of death. Open dialogue regarding matters related to death is imperative for advanced cancer patients. However, the current state of discussing death-related issues in China is less than ideal, and engaging in candid and honest conversations about death and dying has not been well-received [32]. Educating family members about death holds significant importance, yet it remains unpopular in China. Assessing the quality of communication about death between physicians, patients, and patients’ caregivers may currently be inappropriate in China. The deficiency in death education in China is influenced by the traditional taboo surrounding death in Chinese culture [33]. The reluctance or discomfort in discussing death and dying in China signifies a broader challenge in effective communication about end-of-life issues. Healthcare providers may encounter barriers in engaging patients and families in conversations about sensitive topics like death, necessitating tailored approaches to foster open dialogue. Therefore, enhancing public awareness and understanding of end-of-life care through various channels is essential to shift societal perceptions and attitudes towards death. Increased awareness can help normalize discussions about death, empower individuals to make informed decisions, and ultimately improve the quality of end-of-life experiences for patients and their families.

Our findings indicate that a two-factor structure is adequate for both the QOC-P and the QOC-F, albeit with different constituent items compared to those identified by the instrument developers [18]. In the Chinese versions of the QOC-P and QOC-F, items related to disease and treatments are categorized under the general communication skills dimension, whereas in the original instruments, these items fall under the end-of-life care dimension. Within the Chinese cultural context, discussions concerning disease-related specifics such as the treatment plan, rehabilitation, and prognosis are commonly addressed during hospitalization. This may be attributed to the fact that the patients included in our study were undergoing active treatment, leading both patients and their family caregivers to perceive these items as general information. Notably, the items involving communicating feelings about the possibility of the patient deteriorating in the QOC-P and QOC-F were grouped within the end-of-life care dimension. This suggests that even though patients did not explicitly mention death, they exhibited a high degree of sensitivity towards disease progression and end-of-life matters. When physicians communicate their emotions as the patient’s condition worsens, it can trigger patients to ponder the gravity of their situation, potentially redirecting their focus solely towards end-of-life considerations. These results emphasize the necessity for psychosocial support for patients and families dealing with severe illnesses, even in settings where end-of-life conversations may be evaded. Healthcare providers should be equipped to address patients’ emotional needs and concerns across the illness trajectory, not just during end-of-life stages. The modifications made to the items in the Chinese versions of QOC-PF underscore the importance of considering cultural disparities when adapting assessment tools. It indicates that what defines end-of-life care in one culture may be interpreted differently in another, influencing how patients and caregivers react to survey items. Physicians must be mindful of cultural nuances and patient sensitivities to ensure their communication is culturally sensitive and supportive.

Furthermore, we found that some inter-item correlations are less than 0.2 in the Chinese version of the QOC-P, which may be related to the QOC-P being measured in patients with different types of cancer who had different feelings during the survey filling process. Comprehensively considering the results of reliability and validity, and our approximately normally distributed data, we infer that our data have no ceiling and floor effect. However, due to the limitation of our sample size, we hope that future study can expand sample size, expanding population, and selecting representative populations to further explore potential ceiling and floor effect of the Chinese version of the QOC-P and QOC-F.

Significant negative associations were found between anxiety and depression and the total scores of the QOC-P and QOC-F, which indicates that communication between physicians and cancer patients and their family caregivers is crucial to alleviate anxiety and depression in cancer patients. This finding is consistent with findings from studies using the original version of the QOC [34, 35]. In previous research, patients in the intervention group reported greater satisfaction with communication and greater well-being when their physician practiced active listening and negotiation skills [36]. Therefore, it is advisable for physicians to address treatment-related inquiries and end-of-life care aspects for cancer patients and their caregivers using clear and empathetic language, which significantly enhances patients’ psychological comfort. Subsequent research could explore enhancing physician communication skills through diverse methods, such as virtual simulation training, to bolster their communication efficacy.

The SDM-9 is the gold standard for evaluating shared medical decision-making. Significant associations between the QOC-P and shared decision-making as well as the degree of decision regret further highlight the importance of communication quality for medical decisions. The quality of communication between physicians and patients impacted the process of shared decision-making, and the quality of shared decision-making affected the degree of decision regret. Physician-patient communication is foundational to shared decision-making [37]. Physicians provide medical facts and treatment plans, while patients state their treatment preferences and values. Effective communication between physicians and patients regarding medical information and value preferences can empower and promote patients’ participation in shared medical decision-making, which in turn improves patient satisfaction with shared medical decision-making. Instead of providing patients with a large amount of medical information, physicians can use the “ask-tell-ask” approach to improve the effectiveness of shared decision-making and reduce decision regret [38]. Currently, the shared decision-making system is gradually incorporating family participation, thus transforming the physician-patient model of shared decision-making into a physician-patient-family model of shared decision-making that is more in line with the family-centered Chinese society. Therefore, it is practical to translate both the QOC-P and QOC-F into Chinese to facilitate faster and more scientific integration of caregivers into the shared decision-making model. At the same time, it is necessary to strengthen the training of physicians regarding basic medical information, end-of-life care, and communication skills with patients and their family caregivers.

### Limitations

One limitation of this study is its restriction to a single province in southern China, using convenience sampling in clinical settings may limit the generalizability of the research results in other populations. Future investigations could adopt alternative methodologies, such as stratified sampling across national tertiary hospitals, to acquire more diverse and inclusive samples for evaluating the psychometric validation of the Chinese iterations of the QOC-P and QOC-F. Furthermore, although EFA was utilized to reassess the dimensions, CFA was not conducted again due to sample size constraints. Therefore, subsequent research endeavors should endeavor to enlarge the sample size to facilitate CFA in accordance with the model proposed in this study.

### Implications

Despite these limitations, our study offers valuable implications for future research and practice. Transitioning from a physician-patient shared decision-making model to a physician-patient-family shared decision-making model has the potential to enhance the efficacy of shared decision-making and minimize decision regret. The translation of both the QOC-P and QOC-F into Chinese can facilitate the swift and systematic integration of caregivers into the shared decision-making framework. Moreover, it is essential for physicians to undergo communication training to effectively address treatment-related inquiries and end-of-life care aspects, thereby enhancing the psychological well-being of patients and their family caregivers. At a societal level, there is a need to enhance education on death and end-of-life care, as well as to increase public awareness to elevate the standard of communication surrounding these topics and ultimately enhance the quality of end-of-life experiences.

## Conclusion

The Chinese versions of the QOC-P and QOC-F encompass two dimensions: general communication skills and end-of-life communication skills. These questionnaires exhibit acceptable psychometric properties for assessing the quality of communication between physicians and cancer patients along with their family caregivers in China. In the future, we should use more diverse and inclusive samples to re-test CFA based on the structure of the Chinese version of the QOC-P and QOC-F. In both clinical and research settings, the findings of this study can offer valuable insights to aid physicians and researchers in enhancing their understanding and refining the communication quality based on feedback from cancer patients and their family caregivers.

### Electronic supplementary material

Below is the link to the electronic supplementary material.

Supplementary Material 1

## Data Availability

The datasets used and/or analyzed during the current study are available from the corresponding author on reasonable request.

## Abbreviations

COPD: Chronic Obstructive Pulmonary Disease
AIDS: Acquired Immunodeficiency Syndrome
QOC: Quality of Communication
QOC-P: Quality of Communication For Patients
QOC-F: Quality of Communication For Family Caregivers
SEM: Standard Error of Measurement
MCID: Minimal Clinical Important Difference
CVI: Content Validity Index
I-CVI: Item-Content Validity Index
S-CVI: Scale-Content Validity Index
ICC: Intraclass Correlation Coefficient
EFA: Exploratory Factor Analysis
CFA: Confirmatory Factor Analysis
RMSEA: Root-Mean-Square Error Of Approximation
CFI: Comparative Fit Index
PHQ-4: The Patient Health Questionnaire for Depression and Anxiety
DRS: The Decision Regret Scale
SDM-Q-9: The 9-item Shared Decision Making Questionnaire
