# Supplementary material for: Psychometric validation of the Chinese versions of the quality of communication questionnaires for cancer patients and their family caregivers
Source: BMC Nurs. 2024 Jun 20;23:413. doi: 10.1186/s12912-024-02071-z (PMC11188171; doi:10.1186/s12912-024-02071-z)
Supplement: Supplementary file 1 — Supplementary Material 1 [file 12912_2024_2071_MOESM1_ESM.docx]

**Table S1**

**The individual item score of the Chinese version of the QOC-P**

| **Item** | **Mean** | **SD** | **Skewness** | **Kurtosis** |
| --- | --- | --- | --- | --- |
| 1 | 8.44 | 1.265 | –2.585 | 11.737 |
| 2 | 8.38 | 1.234 | –2.344 | 10.510 |
| 3 | 9.12 | 1.258 | –3.927 | 18.613 |
| 4 | 8.67 | 1.105 | –.718 | 1.909 |
| 5 | 8.39 | 1.305 | –2.038 | 10.467 |
| 6 | 8.12 | 1.322 | –1.876 | 7.435 |
| 7 | 8.18 | 1.311 | –.564 | 2.097 |
| 8 | 8.52 | 1.286 | –2.842 | 10.584 |
| 9 | 8.94 | 1.142 | –3.333 | 11.906 |
| 10 | 7.47 | 1.998 | –.825 | –.879 |
| 11 | 7.26 | 1.998 | .160 | –1.662 |
| 12 | 7.47 | 1.945 | –1.274 | .274 |
| 13 | 7.87 | 1.408 | –1.383 | .469 |
| 14 | 7.47 | 1.578 | –.830 | –.864 |
| 15 | 7.63 | 1.471 | –.960 | –.709 |
| Total | 82.40 | 10.33 | –.747 | 2.307 |

**Table S2**

**The individual item score of the Chinese version of the QOC-F**

| **Item** | **Mean** | **SD** | **Skewness** | **Kurtosis** |
| --- | --- | --- | --- | --- |
| 1 | 8.55 | 1.277 | –2.418 | 11.250 |
| 2 | 9.15 | 1.084 | –4.081 | 21.091 |
| 3 | 9.30 | 1.194 | –4.087 | 20.031 |
| 4 | 8.77 | 1.115 | –2.815 | 14.848 |
| 5 | 8.19 | 1.403 | –.508 | 1.259 |
| 6 | 7.74 | 1.543 | –1.351 | 5.235 |
| 7 | 7.70 | 1.549 | –1.371 | 5.141 |
| 8 | 8.48 | 1.184 | –2.878 | 11.646 |
| 9 | 8.49 | 1.174 | –2.660 | 7.000 |
| 10 | 9.04 | 1.037 | –3.304 | 10.953 |
| 11 | 7.60 | 1.184 | –1.371 | .535 |
| 12 | 7.01 | 2.589 | .733 | –1.151 |
| 13 | 8.35 | 1.284 | –2.399 | 6.268 |
| Total | 84.11 | 10.93 | –1.819 | 9.950 |

**Table S3**

**The Chinese version of the QOC-P**

| **Items** |
| --- |
| **General communication skills** |
| 1. Using words that you can understand. |
| 2. Looking into your eyes. |
| 3. Involving your loved ones in decisions about your illness and treatment. |
| 4. Answering all your questions about your illness and treatment. |
| 5. Listening to what you have to say. |
| 6. Caring about you as a person. |
| 7. Giving you his/her full attention. |
| 8. Talking to you about the details of how you might get sicker. |
| **Communication skills about end-of-life care** |
| 9. Talking with you about your feelings regarding the possibility that you might get sicker. |
| 10. Talking to you about how long you might have to live. |
| 11. Involving you in treatment decisions that you want if you get too sick to speak for yourself. |
| 12. Asking about things that are important to you in your life. |
| 13. Respecting things that are important to you in your life. |
| 14. Asking about your spiritual or religious beliefs. |
| 15. Respecting your spiritual or religious beliefs. |

**Table S4**

**The Chinese version of the QOC-F domains**

| **Items** |
| --- |
| **General communication skills** |
| 1. Using words that you can understand. |
| 2. Providing you with information about your loved one’s illness and treatment. |
| 3. Involving you in discussions about the illness and treatment of your loved one. |
| 4. Answering all questions about the illness and treatment of your loved one. |
| 5. (The doctor) Talking with you about the detail that your loved one’s disease. |
| 6. Helping your family in deciding what kind of treatment your loved one wants. |
| **Communication skills about end-of-life care** |
| 7. Listening to what you have to say. |
| 8. Caring about you as a person. |
| 9. Giving you his/her full attention. |
| 10. (The doctor) talking with you about your feelings that your loved one might get sicker or die. |
| 11. When your loved one is able to speak for himself/herself, (the doctor) ask what kind of treatment he/she wants. |
| 12. Asking you about things that are important to your loved one in his/her life. |
| 13. Asking about your spiritual or religious beliefs. |
